# The effects of long-term moderate exercise and Western-type diet on oxidative/nitrosative stress, serum lipids and cytokines in female Sprague Dawley rats

**DOI:** 10.1007/s00394-021-02639-4

**Published:** 2021-07-28

**Authors:** Maria Donatella Semeraro, Gunter Almer, Melanie Kaiser, Sieglinde Zelzer, Andreas Meinitzer, Hubert Scharnagl, Simon Sedej, Hans-Jürgen Gruber, Markus Herrmann

**Affiliations:** 1 Clinical Institute of Medical and Chemical Laboratory Diagnostics, Medical University of Graz, 15/1 Auenbruggerplatz, 8036 Graz, Austria; 2 Department of Cardiology, Medical University of Graz, 8036 Graz, Austria; 3 BioTechMed Graz, 8010 Graz, Austria; 4 Faculty of Medicine, University of Maribor, 2000 Maribor, Slovenia

**Keywords:** Long-term moderate exercise, Western-type diet, Oxidized LDL, Nitric oxide, Nitric oxide synthase, Sprague Dawley rats

## Abstract

**Purpose:**

Regular exercise reduces obesity and the risk of cardiovascular disease. However, health-promoting benefits of physical activity are commonly associated with increased inflammation and oxidative stress. Here, we tested whether constant moderate exercise is able to prevent or attenuate the oxidative/nitrosative stress, inflammation, and serum lipids in lean and obese rats.

**Methods:**

Four-month-old female Sprague Dawley rats received standard or a high-fat diet. Animals were subjected to a physical activity protocol, consisting of 30 min forced treadmill exercise for 5 consecutive days per week during 10 months. Baseline and sedentary (non-exercised) rats were used as controls. Lipids, oxidized low-density lipoprotein cholesterol, nitric oxide metabolites, and pro- and anti-inflammatory markers were measured in blood collected upon euthanasia.

**Results:**

At variance to young baseline control rats, 14-month-old animals fed normal diet had increased plasma lipid levels, including total cholesterol and triglycerides, which were further elevated in rats that consumed a high-fat diet. While treadmill exercise did not lower the amount of serum lipids in standard diet group, forced physical activity reduced non-high-density lipoprotein cholesterol in response to high-fat diet feeding. Exercised rats fed standard diet or high-fat diet had lower abundancy of nitric oxide metabolites, which coincided with increased levels of oxidized low-density lipoprotein cholesterol. Accordingly, the amount of nitric oxide metabolites correlated inversely with oxidized low-density lipoprotein cholesterol and homo-arginine. Exercise significantly reduced inflammatory cytokines in high-fat diet fed rats only.

**Conclusion:**

Our study suggests that regular exercise alters the equilibrium between oxidative and anti-oxidative compounds and reduces pro-inflammatory cytokines.

## Introduction

Cardiovascular disease (CVD) is a major cause of morbidity and mortality in Western societies and developing countries [1]. Modifiable risk factors, such as obesity and sedentarism are highly prevalent in patients with CVD, and both can be improved by safe and effective lifestyle interventions [2]. Such lifestyle factors are diet and exercise that affect the concentration of low-density lipoproteins (LDL), which are known metabolic driver of CVD, such as atherosclerosis [3–5]. In addition, it is well established that elevated concentrations of LDL cholesterol (LDL-C) promote atherosclerosis and increase the risk of non-fatal and lethal CVD events. LDL-C is subjected to oxidation, resulting in the formation of oxidized LDL (oxLDL), which is thought to be more aggressive than non-oxidized LDL in inducing atherogenesis [6, 7].

Elevated oxidative stress characterized by an increased generation of radical oxygen species (ROS) is a common manifestation in patients with CVD, obesity, and diabetes mellitus. These highly reactive compounds can modify many biomarkers including LDL-C [8–10]. The modification of LDL-C by oxidation promotes the receptor-mediated uptake of oxLDL by macrophages, thereby causing cholesterol accumulation in the vasculature. Previous studies in humans and animals indicate that Western diet and physical inactivity induce free radical production, which may enhance the susceptibility of LDL to oxidation [3, 4, 11]. One of the principal pathways involved in the adaptation to exercise is nitric oxide (NO) signalling. NO triggers vasodilation and, thus, can mitigate the high shear stress during exercise. Furthermore, NO is involved in several other physiological and pathological processes, such as cell inflammation and adhesion as well as angiogenesis [12–15]. NO is synthetised by three isoforms of the enzyme NO synthase (NOS), including neuronal (nNOS or NOS-1), inducible (iNOS or NOS-2), and endothelial isoform (eNOS or NOS-3). All of them are constitutively but not exclusively expressed in the cardiovascular system [10]. In particular, eNOS requires dimerization to maintain its normal function but under certain conditions the reduction of molecular oxygen by eNOS is not coupled anymore with the oxidation of the substrate L-arginine (L-arg), thus resulting in the production of superoxide instead of NO [10]. The main eNOS uncoupling motifs are the cofactor of NO synthesis, tetrahydrobiopterin (BH4), reduced bioavailability of L-arg, or high levels of the endogenous NOS inhibitor, asymmetrical dimethyl-arginine (ADMA) [8].

Although regular exercise is proven to reduce CVD risk and promote health benefits [12, 16–19], constant physical activity also increases the production of free radicals and oxidative stress [4, 9]. This raises the question as to whether exercise-induced oxidative stress is beneficial or detrimental. In this regard, obese and normal-weight human adults have reportedly comparable oxLDL concentrations [20], whereas other studies showed that body weight reduction after the bariatric surgery or regular moderate exercise decrease oxLDL [21, 22]. In contrast, a single intensive exercise session appears to increase the susceptibility of LDL and other lipoproteins to oxidation in healthy adults [3, 4]. Thus far, a single in vivo study explored the effects of HFD and regular physical activity on plasma lipids, oxidative/nitrosative stress and LDL oxidation. Specifically, Elmas et al. showed that rats consuming a HFD exhibit increased oxidative stress in aortic and myocardial tissue [2]. Regular exercise appears to modify the balance of antioxidants and oxidants as well as NO metabolism in these tissues. However, it remains elusive whether constant moderate exercise is able to prevent or attenuate the oxidative/nitrosative stress, inflammation, and serum lipids in lean and obese rats.

In the present study, we investigated the impact of HFD and regular moderate exercise on the oxidative/nitrosative stress, serum lipids and cytokines. For this purpose, blood samples from young and old Sprague Dawley female rats that were subjected to forced treadmill exercise sessions for 10 months in combination with a HFD or ND were analysed.

## Materials and methods

### Animal model

Four-month-old healthy female SD rats (*n* = 120) with an average body weight of approximately 300 g were purchased from Janvier Labs (France) and kept in groups of three animals per cage under constant conditions on a 12 h light and 12 h dark cycle in the institutional animal facility. The decision to work with female animals aimed to avoid gender effects and to reduce the risk of dropouts due to aggressive behaviour between animals. After 1 week of acclimatization, the animals were randomly assigned to receive a standard diet (ND) (Altromin, Germany) with 3.23 kcal/kg and 11% fat or a custom-designed beef-tallow high-fat diet (HFD), rich in saturated fatty acids (SFA), in particular C16:0 and C18:0, with 5.15 kcal/kg and 60% fat (Table 1; ssniff, Germany). Saturated fatty acids (SFA) and mono-unsaturated fatty acids (MUFA) are present in a ratio of 1:1. While the ratio of SFA and poly-unsaturated fatty acids (PUFA) is close to 5:1. The HFD composition was based on previous studies [23–25]. Food and tap water were provided ad libitum.

**Table 1:** Composition of the high-fat diet

Fatty acids		Vitamins	
Saturated fatty acids	%	Antioxidant	Per kg
C 12:0	0.04	Vitamin A	15,000 IU
C 14:0	1.18	Vitamin E	150 mg
C 16:0	8.27		
C 17:0	0.38	Others	
C 18:0	6.06	Vitamin D_3_	1500 IU
C 20:0	0.04	Vitamin K (as MNB)	20 mg
		Thiamine (B_1_)	25 mg
Mono-unsaturated fatty acids		Riboflavin (B_2_)	16 mg
C 16:1	1.33	Pyridoxine (B_6_)	16 mg
C 18:1	12.29	Cobalamin (B_12_)	30 µg
		Nicotinic acid	47 mg
Poly-unsaturated fatty acids		Pantothenic acid	55 mg
C 18:2	2.53	Folic acid	16 mg
C 18:3	0.34	Biotin	300 µg
		Choline	920 mg

Fatty acids and antioxidant vitamins content

### Experimental design and treatment

Animals were randomly allocated to 5 groups, each consisting of 24 animals. The rats in group 1 were sacrificed after the acclimatization period and served as a baseline control (BL). Ninety-six animals were divided in a 1:1 ratio, fed ND or HFD and subjected to the 10-month study protocol as follows: half of the rats fed ND or HFD performed 30-min running exercise sessions on a treadmill (Panlab, Spain) on 5 consecutive days (indicate at what time of the day) followed by 2 days of rest. The running speed was constant and set at 30 cm/s. The training protocol was based on previous experimental studies [26–29]. The animals that did not exercise throughout the entire study period were used as sedentary controls.

### Euthanasia and sample preparation

At the end of the 10-month study period, blood was drawn by heart puncture under deep isoflurane anaesthesia (Forane, Abbott, Austria). Also for the baseline animals, the blood was collected only once at the time of sacrification. Blood and plasma were collected using S-Monovette Serum-Gel tubes and S-Monovette Plasma-EDTA tubes (Sarstedt, Nümbrecht, Germany), respectively. Samples were centrifuged at 2000 g for 12 min at room temperature, aliquoted and stored at − 80 °C until the analysis. Blood collections and consequently serum analyses were performed in a non-fasting state.

### Evaluation of the oxidative/nitrosative stress

The circulating oxLDL concentration was determined in serum (100 µl) with a rat-specific commercial Sandwich ELISA kit (USCN Life Sciences, Texas) according to the manufacturer’s instructions. This assay uses rat polyclonal antibodies against oxLDL and has a measurement range between 31.2 and 2000 pg/ml. In this range, intra- and inter-assay imprecision is below < 12%. NO was estimated in serum (100 µl) by measuring the degradation products nitrite (NO_2_^−^) and nitrate (NO_3_^−^) using a commercial photometric method (NO quantification kit, Active Motif, California) on a FlexStation3 (Molecular devices, California). In addition, plasma concentrations of homo-arginine (h-arg), ADMA and symmetrical dimethyl-arginine (SDMA) were quantified by a reverse-phase high-pressure liquid chromatography (HPLC) method as described previously [30, 31].

### Assessment of systemic inflammatory markers

A profile of 22 inflammatory markers and chemokines, including Regulated and Normal T-cell Expressed and Secreted (RANTES), eotaxin, macrophage inflammatory protein 1α (MIP-1 *α*), monocyte chemoattractant proteins 1 and 3 (MCP-1 and 3), tumour necrosis factor α (TNF-*α*), interferon *γ* (IFN-*γ*), IFN-*γ*-inducible protein (IP-10), and interleukins (IL-1β, IL-2, IL-5, IL-6, IL-10, IL-12, IL-17) were determined in 25 µl of serum with a preconfigured multiplex immunoassay kit (ThermoFisher Scientific, Austria) using the BioPlexTM 200 detection system (Bio-Rad, Austria). The activity of matrix metalloproteinases (MMPs) was measured through an enzymatic reaction using a Mca-PLGL-Dpa-AR-NH_2_-fluorogenic peptide substrate (R&D Systems, Canada). Serum (90 µl) was incubated with the diluted working solution (10 µl) for 20 min at room temperature. The fluorescent signal was detected at 320 nm excitation and 405 emission wavelength using the photometer FLUOstar OPTIMA (BMG Labtech, Germany).

### Assessment of lipid metabolism and adipocytokines

The serum lipid profile was determined on fully automated Olympus AU640 analyser (Olympus, Hamburg, Germany) using commercial assays. Briefly, total cholesterol (TC), triglycerides (TG), phospholipids (PL), non-esterified fatty acids (NEFA), and HDL cholesterol (HDL-C; homogeneous assay) were measured using enzymatic methods and reagents from Diasys (Holzheim, Germany). The instrument was calibrated using secondary standards from Roche Diagnostics (Mannheim, Germany; for TC, TG) and Dyasis (Holzheim, Germany; for FC, PL). Insulin growth factor-1 (IGF-1), leptin and adiponectin were evaluated in serum by commercial sandwich ELISAs (Demeditec Diagnostics Gmbh, Germany) according to the manufacturer’s instructions. Finally, resistin was quantified using a sandwich enzyme immunoassay from BioVendor—Laboratorní medicína a.s. (Brno, Czech Republic).

### Statistical analyses

Data are presented as means ± standard deviations. The differences between groups were assessed using two-tailed Student’s *t* test for dependent or independent samples or Mann–Whitney *U* test depending on the distribution of the data. Correlations between variables were determined by linear regression analysis according to Pearson (*r*, Pearson correlation coefficient; *p*, univariate ANOVA). *p* value of < 0.05 was considered statistically significant. Analyses were performed by explorative data analyses using SPSS for Windows (IBM^©^ SPSS^©^ Statistics, version 25).

## Results

### Exclusion criteria and body weight

**Fig. 1:**
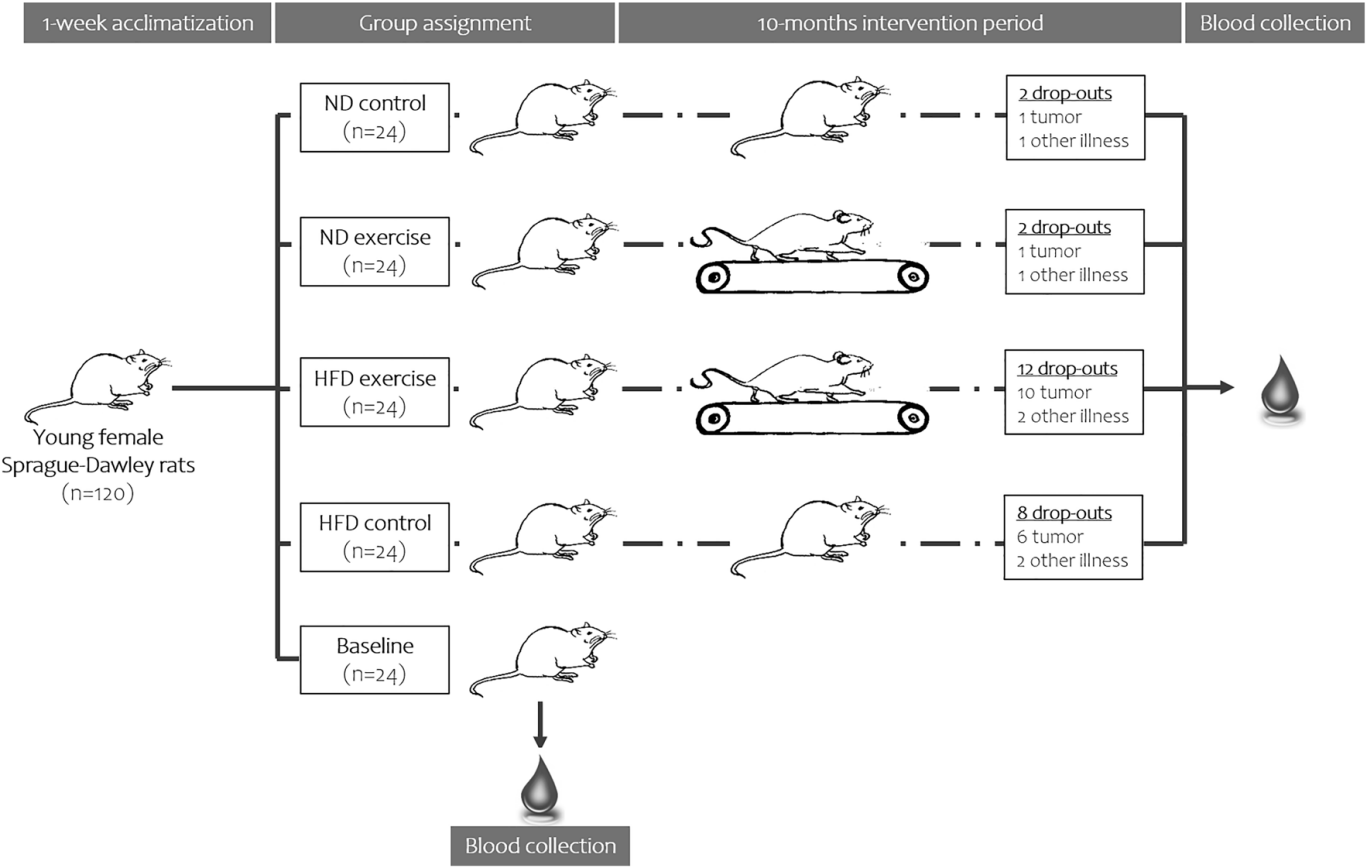
The experimental design. One-hundred and twenty young female Sprague Dawley rats were randomly allocated into 5 groups, each consisting of 24 animals. The animals in the baseline control group were euthanized after the acclimatization period. The remain of ninety-six animals were divided in a 1:1 ratio and fed ND or HFD and subjected to a 10-month study protocol as follows: half of the rats fed ND or HFD performed 30-min running exercise sessions on a treadmill. Six animals (2 vs. 4 rats fed ND and HFD, respectively) died prior to the end of the study and eighteen more (2 vs. 16 rats under ND and HFD) had to be excluded due to the development of tumours

**Fig. 2:**
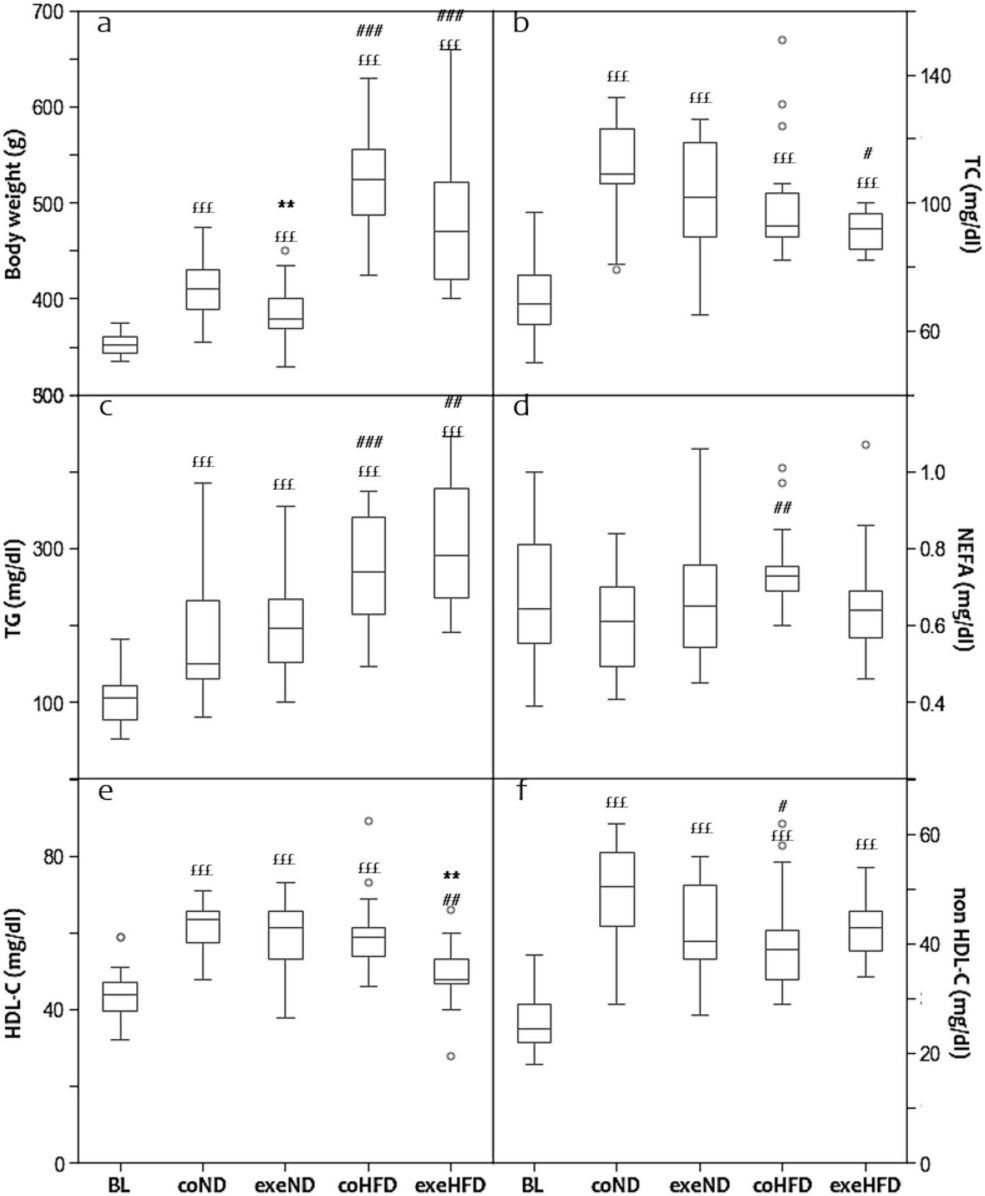
Box and Whisker Blot of the body weight (**a**) and the lipid profile (**b**–**f**) after the 10 months study period. **p < *0.01* compared to appropriate sedentary control group; ^#^*p* < *0.05,* ^##^*p* < *0.01,* ^###^*p* < *0.001* compared to appropriate normal diet control group; ^*£££*^*p* < *0.001* compared to baseline control group. The study groups have been abbreviated as follows: baseline group (BL), control normal diet (coND), exercise normal diet (exeND), control high fat diet (coHFD) and exercise high-fat diet (exeHFD)

From the 120 rats included in the study, 6 rats had to be sacrificed before the end of exercise protocol because of illness, while additional 18 animals developed tumours and, thus, were excluded from the analysis (Fig. 1) [32]. Tumours were more frequent in animals fed high-fat than standard diet (16 vs. 2 rats). At the end of the study, 96 animals were included in the analysis. All animals had significantly increased body weight as compared to the baseline group (Fig. 2a, *p* < *0.001*). However, this increase in body weight was markedly higher in response to HFD than ND feeding. Also organ weights increased with age and HFD (Table 2).

**Table 2:** Organ weight and serum biomarkers in female Sprague Dawley rats after 10 months of treadmill exercise

	Baseline *n* = 24	Normal diet group			High-fat diet group	
		Control *n* = 22		Exercise *n* = 22	Control *n* = 16	Exercise *n* = 12
Organ weight						
Heart						
Average weight	1.18 ± 0.12	1.31 ± 0.21^£^		1.24 ± 0.11	1.40 ± 0.14^£££^	1.46 ± 0.19^###£££^
Normalized weight	0.26 ± 0.03	0.28 ± 0.04^£^		0.27 ± 0.03	0.30 ± 0.03^£££^	0.31 ± 0.03^###£££^
Liver						
Average weight	2.39 ± 0.25	12.53 ± 1.72^£££^		12.50 ± 1.80 ^££^	14.03 ± 2.36^#£££^	15.16 ± 4.40^#£££^
Normalized weight	2.39 ± 0.25	2.67 ± 0.31^££^		2.56 ± 0.68	2.98 ± 0.52^#£££^	3.28 ± 0.89^#£££^
Visceral fat						
Average weight	5.81 ± 2.07	13.20 ± 5.26^£££^		10.46 ± 4.48^£££^	40.13 ± 12.81^###£££^	39.46 ± 23.20^###£££^
Normalized weight	0.01 ± 0.00	0.02 ± 0.01^£££^		0.02 ± 0.01^£££^	0.07 ± 0.02^###£££^	0.07 ± 0.04^###£££^
Biochemical analyses						
SDMA	0.43 ± 0.08	0.27 ± 0.11^£££^		0.28 ± 0.08^£££^	0.33 ± 0.04^#£££^	0.20 ± 0.04***^##£££^
Resistin	14.97 ± 5.15	8.16 ± 3.45^£££^		7.72 ± 2.94^£££^	7.78 ± 3.86^£££^	7.49 ± 3.99^£££^
Eotaxin (CCL11)	230.53 ± 46.15	420.79 ± 104.54^£££^		335.76 ± 94.67**^£££^	372.79 ± 143.41^£££^	294.85 ± 150.66
MIP-1α (CCL3)	18.42 ± 5.05	18.96 ± 5.06		22.47 ± 9.27	25.68 ± 16.33	17.66 ± 4.07*
IFN-γ	38.36 ± 11.58	46.20 ± 31.04		44.22 ± 28.37	42.11 ± 22.80	53.36 ± 39.07
IP-10 (CXCL10)	109.25 ± 33.01	153 ± 35.70^£££^		163.78 ± 46.04^£££^	125.05 ± 51.60	118.30 ± 23.12^##^
IL-5	79.43 ± 29.89	31.59 ± 17.50^£££^		38.57 ± 18.05^£££^	43.41 ± 17.29 ^#£££^	37.27 ± 21.91*^£££^
IL-6	< 0.001	< 0.001		< 0.001	< 0.001	< 0.001
IL-10	< 0.001	< 0.001		< 0.001	< 0.001	< 0.001
IL-12	58.62 ± 17.45	58.07 ± 27.91		121.53 ± 83.54**^£££^	52.18 ± 25.04	45.72 ± 21.98
IL-17	21.79 ± 8.02	18.73 ± 11.90		22.45 ± 15.95	11.28 ± 3.91^£££^	16.37 ± 11.40
MMPs activity	10,881 ± 1177	12,136 ± 1488^££^		14,092 ± 1832***^£££^	11,876 ± 2026	9966 ± 2943^###^

The organ weight is given in grams; organ weight is normalized to total tibia length (cm). SDMA is expressed in µM, while resistin in ng/ml. All other cytokines and chemokines are expressed in pg/ml. Data are presented as mean ± standard deviation

*p < *0.05*

**p < *0.01*

***p < *0.001* compared to appropriate sedentary control group

^*#*^*p* < *0.05*

^*##*^*p* < *0.01*

^*###*^*p* < *0.001* compared to the appropriate normal diet control group

^£^*p* < *0.05*

^*££*^*p* < *0.01*

^*£££*^*p* < *0.001* compared to the baseline control group

The exercise protocol was well tolerated in all animals and reduced the weight gain in the ND animals (*p* < *0.01*), but not in HFD animals. Moreover, regular moderate exercise induced an increase of myocardial and hepatic weight in HFD animals, but not in ND animals.

### Lipid profile

Serum lipids are established risk factors of atherosclerosis and CVD that are affected by lifestyle factors, such as diet and physical activity [3–5]. Therefore, we first explored the effects of HFD and exercise on serum lipids. After 10 months of intervention, average TC and TG concentrations were significantly higher compared to baseline, regardless of diet and exercise (Fig. 2b, c). HFD animals showed additional alterations of the lipid profile beyond simple age-related changes. TG and NEFA were significantly higher than in ND animals, whereas non-HDL-C was lower (Fig. 2c, d, f). Exercise induced a decrease of TC and HDL-C in HFD, but not ND animals (Fig. 2b, e). All other lipid parameters were comparable between sedentary and exercising animals in both diet groups.

In an attempt to explore potential mechanism that mediate the changes in plasma lipids, correlation analyses have been performed. At study end, body weight was positively correlated with TG (*r* = 0.598; *p* < *0.001*), NEFA (*r* = 0.238; *p* = *0.025*) and non-HDL-C (*r* = 0.271; *p* = *0.010*), whereas TC (*r* =  − 0.291; *p* = *0.006*) and HDL-C (*r* =  − 0.365; *p* < *0.001*) were inversely correlated to adiponectin.

### Oxidative/nitrosative stress

**Fig. 3:**
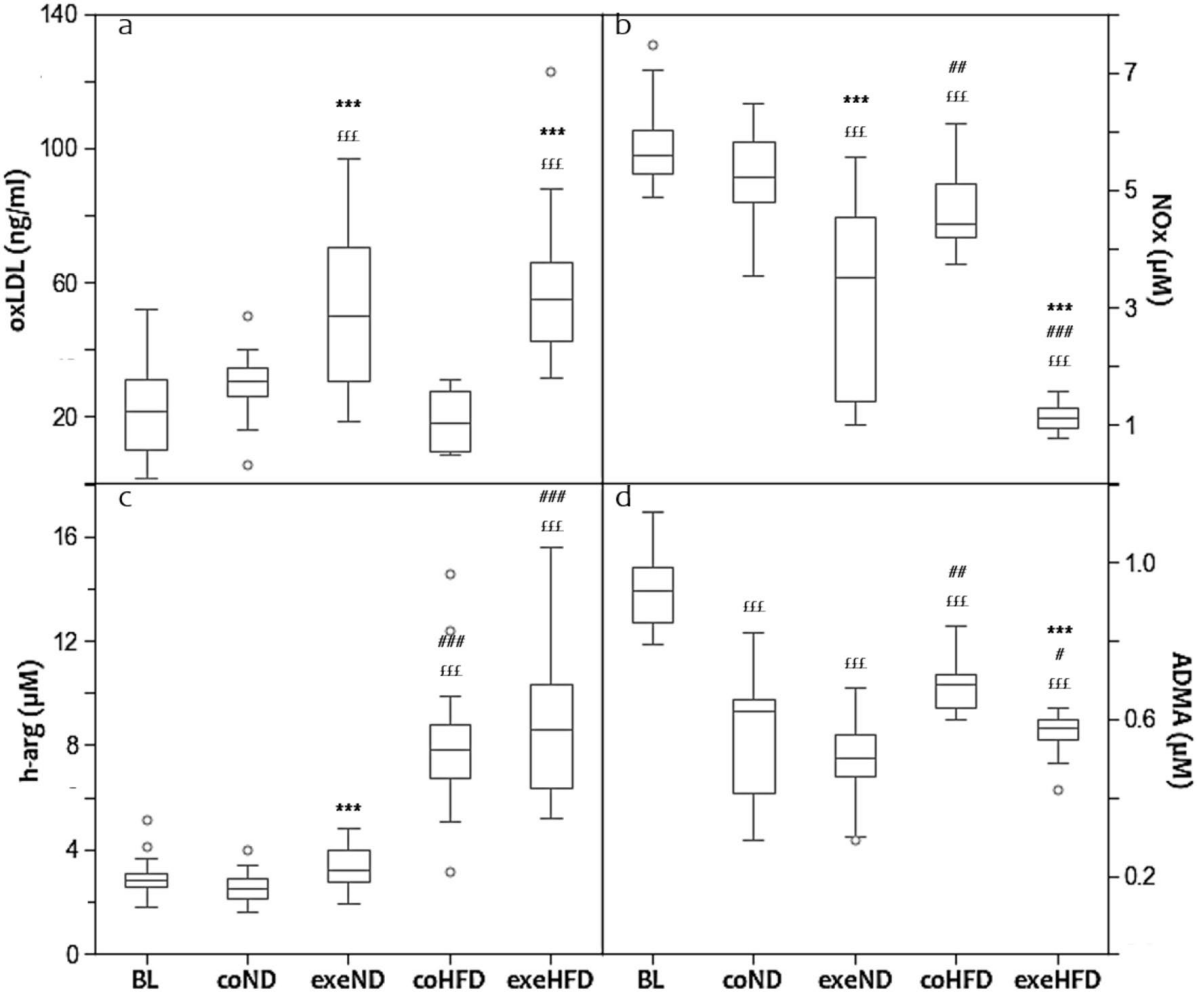
Box and Whisker Blot of oxidized LDL (**a**). ***p < *0.001* compared to appropriate sedentary control group; ^*£££*^*p* < *0.001* compared to baseline control group. Box and Whisker Blot of nitric oxide metabolites (**b**). ***p < *0.001* compared to appropriate sedentary control group; ^*##*^*p* < *0.01*, ^*###*^*p* < *0.001* compared to appropriate normal diet control group; ^*£££*^*p* < *0.001* compared to baseline control group. Box and Whisker Blot of homo-arginine and ADMA (**c**–**d**). ***p < *0.001* compared to appropriate sedentary control group; ^*#*^*p* < *0.05*, ^*##*^*p* < *0.01* compared to appropriate normal diet control group; ^*£££*^*p* < *0.001* compared to baseline control group

Advanced age, obesity and physical inactivity are known to modulate the equilibrium between oxidant and antioxidant compounds, with consequent alterations of lipid peroxidation and NOS function [8–10]. Therefore, we analysed the effects of HFD and exercise on oxLDL and NOx. Figure 3 shows that age and diet had a substantial effect neither on oxLDL nor on NOx (Fig. 3a, b). Exercise instead altered both biomarkers significantly. After 10 months of regular training, oxLDL was increased in ND and HFD by 44% and 68%, respectively. In contrast, NOx was markedly lower in exercising animals with the lowest concentrations in the HFD group.

To corroborate the NOx results, we also measured the non-proteogenic amino acid h-arg, a substrate of NOS for the production of NO, and ADMA, a competitive inhibitor of NOS. h-arg was significantly higher in HFD animals than in ND and baseline controls (Fig. 3c). Exercise increased h-arg in ND animals, but not in HFD animals. The serum concentrations of ADMA were highest at baseline and decreased with age. This decrease was less pronounced in HFD than in ND animals. Exercise reduced ADMA in the HFD group, but not in the ND group (Fig. 3d, *p* < *0.001*).

**Fig. 4:**
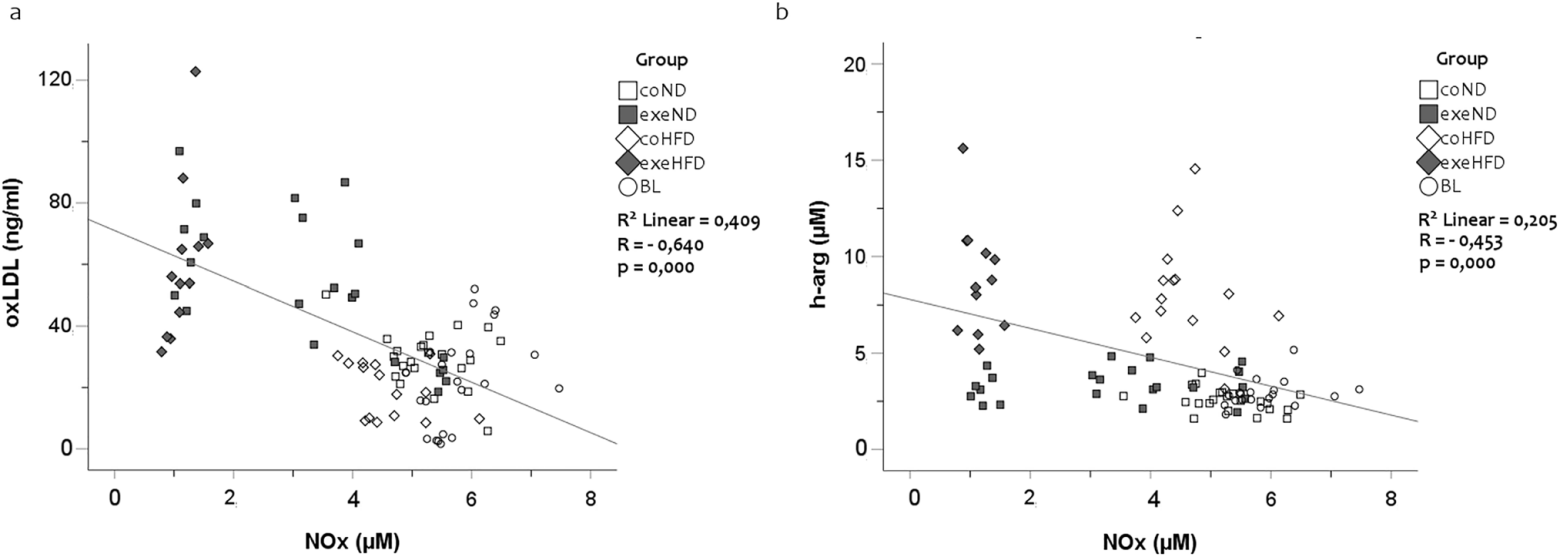
Simple Scatter Dot Plot of the linear regression analysis with *r* = − 0.640 and *p* = *0.000* between oxidized LDL-C and nitric oxide metabolites (**a**) and *r* = − 0.453 and *p* = *0.000* between h-arg and nitric oxide metabolites (**b**)

Considering that oxidative stress and NOS function are linked with each other, we performed linear regression analyses that showed a strong inverse association between oxLDL and NOx (Fig. 4a). An inverse relationship was also found between h-arg and NOx (Fig. 4b, *r* = − 0.453; *p* < *0.001*). ADMA and SDMA were positively related to NOx with (ADMA *vs.* NOx, *r* = 0.401; *p* < *0.001*) and (SDMA *vs.* NOx, *r* = 0.459; *p* < *0.001*), but inversely associated with oxLDL with (*r* = − 0.338; *p* < *0.001*) and (*r* = − 0.274; *p* = *0.009*), respectively.

### Effects of physical activity and diet on cytokines and chemokines

**Fig. 5:**
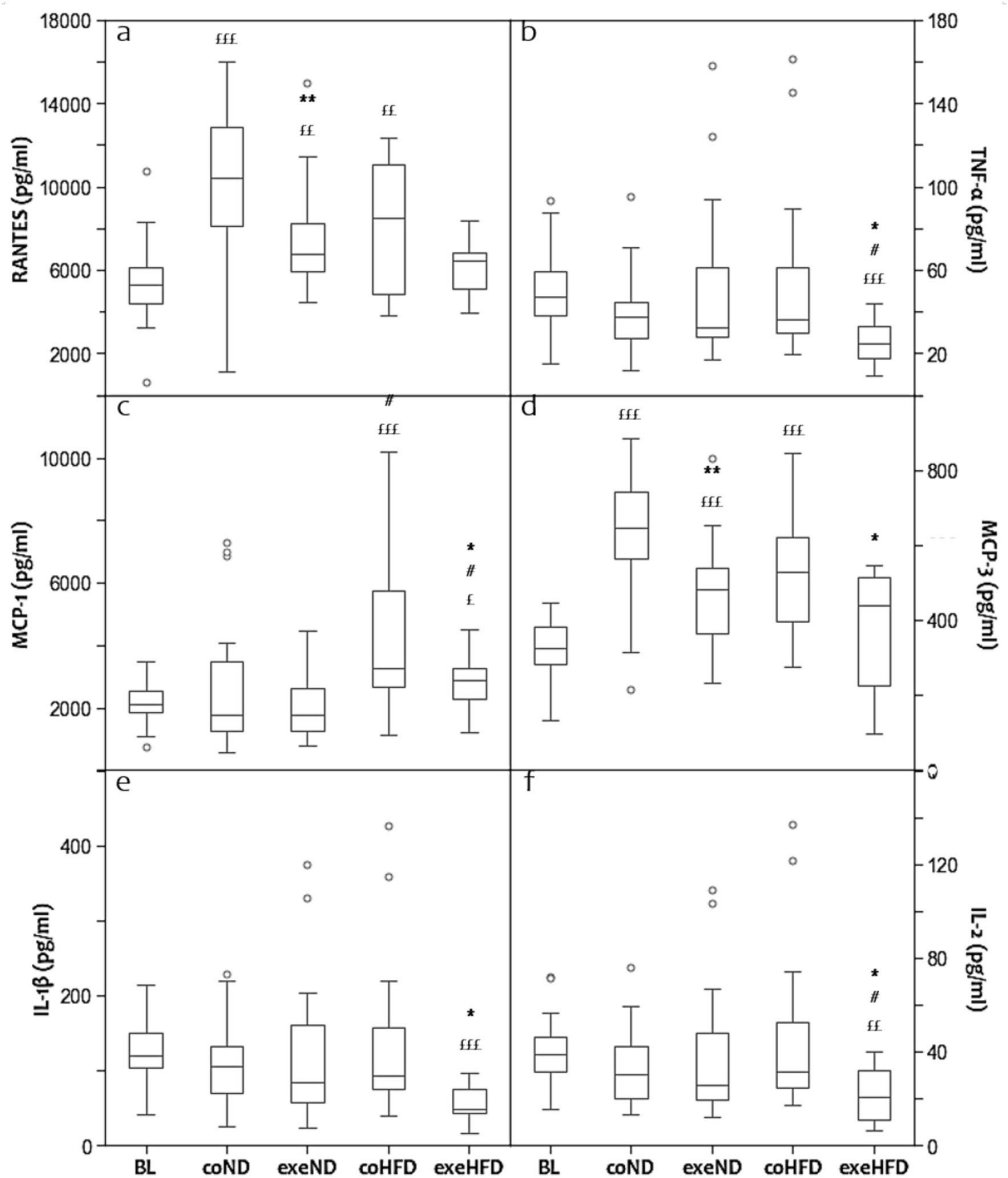
Box and Whisker Blot of the panel of cytokines and chemokines (**a**–**f**). *p < *0.05*, **p < *0.01* compared to appropriate sedentary control group; ^*#*^*p* < *0.05* compared to appropriate normal diet control group. ^*£*^*p* < *0.05*, ^*££*^*p* < *0.01*, ^*£££*^*p* < *0.001* compared to baseline control group

Dyslipidaemia and oxidative/nitrosative stress are established drivers of chronic systemic inflammation, an important factor in the pathogenesis of atherosclerosis [6, 7]. To study the immunological response of HFD and physical activity, we analysed a broad panel of pro- and anti-inflammatory cytokines (Fig. 5). Regular treadmill exercise reduced the serum concentrations of the pro-inflammatory cytokines, such as TNF-α, IL-1β and IL-2 in rats consuming HFD, but not those fed ND (Fig. 5b, e, f). All other cytokines were not significantly affected by exercise. Age and diet alone had no significant effects on the serum concentrations of the measured pro-and anti-inflammatory cytokines (Table 2).

Chemokines are secreted signalling proteins that mediate the migration of immune cells in response to pro-inflammatory cytokines [33]. In the present study, several pro-inflammatory chemokines, including RANTES, MCP-1 and 3, were measured. In 14-month-old sedentary ND and HFD animals, the average serum concentrations of RANTES and MCP-3 were significantly higher than in young baseline controls (Fig. 5a, d). Regular exercise attenuated this age-related increase irrespective of diet. MCP-1 showed similar trends, but due to a greater inter-individual variability of this marker, significant effects were present only in HFD animals (Fig. 5c).

### Adipocytokines

**Fig. 6:**
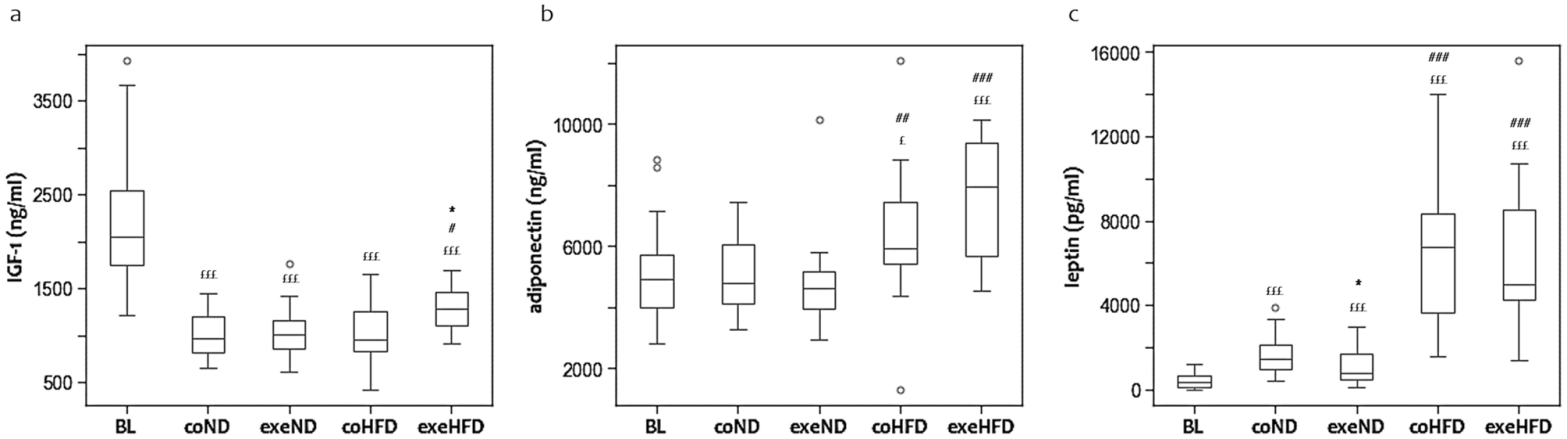
Box and Whisker Blot of IGF-1 (**a**), adiponectin (**b**) and leptin (**c**). *p < *0.05* compared to appropriate sedentary control group; ^*#*^*p* < *0.05, *^*##*^*p* < *0.01, *^*###*^*p* < *0.001* compared to appropriate normal diet control group. ^*£*^*p* < *0.05*, ^*£££*^*p* < *0.001* compared to baseline control group

Adipocytokines, such as adiponectin, leptin, and IGF-1 are key regulators of energy metabolism and fat stores that are centrally involved in the pathomechanistic sequaele of adipositas and obesity [34, 35]. Furthermore, they have immune-modulatory effects [36]. In the present study, age, diet, and exercise had profound effects on the serum concentrations of IGF-1, adiponectin, and leptin (Fig. 6). At the end of the 10-month protocol, IGF-1 concentration was approximately 50% lower than in young baseline controls, irrespective of diet (Fig. 6a). Exercising animals fed HFD, had slightly higher IGF-1 concentrations than their respective controls. In contrast, leptin was markedly higher in old than in young animals (Fig. 6c). The age-related increase in serum leptin was substantially amplified by HFD. Exercise reduced serum leptin concentrations slightly in both diet groups. Adiponectin was not affected by age and exercise in ND animals, but increased in HFD animals (Fig. 6b). This increase was greater in exercising HFD animals. Resistin decreased with age in HFD and ND animals (*p* < *0.001*). In both dietary groups, exercising animals had even lower resistin serum concentrations than sedentary counterparts.

## Discussion

The present study shows that long-term moderate exercise reduces the body weight gain and NOx but increases oxLDL in normal-weight and obese rats. Administering HFD to sedentary animals resulted in a marked increase in body weight gain and triglycerides; however, it failed to systematically alter oxLDL, NOx or any other lipid profile parameter.

In the SD rats used in this project, long-term moderate running exercise did not change TC, HDL-C, and non-HDL-C. In HFD fed animals, TG was slightly reduced by exercise. Furthermore, exercise increased oxLDL in both dietary groups. These results are in contrast to most previous studies in humans and rodents where regular moderate endurance exercise reduced TG, TC and LDL-C, whereas HDL-C increased [2, 26, 37–39]. However, the results of individual studies may vary substantially [4, 37, 40] and comparability between studies is limited by different experimental settings. For example, Elmas et al. analysed the effects of a 6-week exercise intervention in SD rats where animals were forced to swim for 1 h/d on 5 days/week [2]. Animals that received a high-fat diet showed a decrease in TC and TG when compared to sedentary controls. In our study, the administration of a HFD increased TG, but not TC. Furthermore, exercise did not show a modulatory effect on TG, TC, or non-HDL-C. When compared to the present results, the lipid concentrations reported by Elmas et al. are quite different. These contrasting results may be explained the use of male animals that were considerably younger at the time of sacrification. Furthermore, the present study used a HFD with a different lipid composition and a less vigorous exercise intervention. Analytical differences may also have contributed to the different results. In our laboratory, all methods are strictly controlled by internal and external quality controls. Furthermore, the lipid concentrations that we obtained in young and old control animals are in line with a previous study in SD rats [11].

The HFD used in this study is particularly rich in SFA and MUFA and thus induces an imbalance of lipoprotein metabolism with consequent alterations of plasma lipoproteins. According to the manufacturer, this diet aims to mimic the situation in humans who follow a typical Western-type dietary regimen and live in an obesogenic environment. Furthermore, it reliably induces obesity and metabolic syndrome in mice and rats. Differences in the dietary lipid composition may explain differences in plasma lipoproteins and oxidative–nitrosative stress markers observed in other HFD intervention studies in rodents [2, 23, 41, 42]. The current National Cholesterol Education Program (NCEP) and American Heart Association (AHA) dietary guidelines recommend limiting fat intake to 30–40% of the total dietary calories [43]. However, increasing fat intake inside the recommended range may already have adverse effects on the lipid profile with increasing TC and LDL-C concentrations [43]. In contrast, reducing fat intake to 20% or less can also be troublesome due to a reduction of LDL-C and HDL-C and a contemporary rise in TG. This combination is typically associated with the formation of small and dense LDL particles with a high atherogenic potential [43]. With the aim to promote a healthier LDL/HDL ratio, the original AHA Step I fat recommendation advices for a 1:1:1 proportion of SFA: MUFA: PUFA in the diet.

In old animals the lipid profile was substantially different from that of young animals with markedly higher TC and TG concentrations. Moreover, HDL-C, non-HDL-C and NEFA were higher in old than in young animals. Such age-related changes of the lipid profile are expected and have been described by others before [44]. With the HFD used in this study, the concentrations of TG and NEFA increased, whereas all other parameters of the lipid profile remained unchanged. Previous studies that treated rodents with HFD reported mixed results. While some studies found increasing concentrations of TC, TG and LDL-C [2, 25, 41, 44] others did not [11]. However, a direct comparison of these studies is limited due to differences in study design and composition of the diets. For example, in 4–8-week-old Wistar rats the administration of HFD for 4–8 weeks resulted in increased body weight and adipose tissue weight, TC, TG, LDL-C concentrations [23, 42]. However, both studies did not include baseline measurements, which impedes a longitudinal evaluation of age-related effects. Zelzer et al. treated adult female SD rats for 12 weeks with a HFD comparable to the one used in the present study [11]. This intervention did not result in different TC or TG concentrations. Also, HDL-C and non-HDL-C were comparable between controls and HFD-treated animals. The limited comparability of different animal models is not surprising and has already been shown before [44]. Similar to animal models, also human studies that compared the lipid profile of obese and non-obese individuals yielded heterogeneous results [20, 21, 45]. Most existing studies showed higher TC, TG, LDL-C, and small dense LDL-C (sdLDL-C) concentrations in obese individuals when compared to normal weight controls. However, for HDL-C, inconsistent results have been reported [21, 45]. A controlled dietary intervention study by Egert et al. demonstrated that the substitution of a high-fat diet rich in saturated fatty acids with either a high-fat or a low-fat diet rich in mono-unsaturated fatty acids ameliorated the lipid profile, reducing TC, LDL-C/HDL-C ratio, LDL-C size and its susceptibility to oxidation [46].

A main finding of our study is the exercise-induced increase in oxLDL that is accompanied by a reduction of NO. Elevated oxidative stress is a common condition in sedentary and obese individuals that increases the generation of ROS and leads to the modification of many biochemical targets [8–10] including LDL-C. OxLDL is supposed to be more aggressive than non-oxidized LDL in driving inflammation, atherogenesis, and ultimately the risk of CVD events [5–7]. Nevertheless, exercise-induced ROS production seems to protect cells against oxidation by maintaining the cellular oxidant-antioxidant homeostasis [47, 48]. Furthermore, regular physical activity improves blood pressure control through an increased production of NO and other vasoactive substances [10]. In the present study, however, long-term moderate running exercise was associated with an increase in oxLDL and a reduction in NOx. While the increase in oxLDL was comparable in both dietary groups, the reduction in NOx was more pronounced in HFD animals. These results are in agreement with previous studies demonstrating an increased susceptibility of LDL-C and other lipoproteins to oxidation after a single intensive exercise session [3, 4]. Furthermore, in another study by Zelzer et al., the administration of a similar HFD to rats increased several markers of oxidative stress, such as malondialdehyde, but not oxLDL [11]. While Zelzer et al. measured oxLDL by ELISA, the other studies analysed lipoprotein oxidation indirectly [3, 4], which limits the comparability of results. When interpreting the modification of oxidative stress biomarkers by physical activity, it should be considered that chronic exercise as well as high-intensity training can increase oxidative stress through several mechanisms including increased mitochondrial oxygen consumption and activation of oxidase enzymes such as nicotinamide adenine dinucleotide phosphate (NADPH) oxidase, a major source of ROS [9]. This might at least partially explain the exercise-induced increase in oxLDL that we observed in the present study. Additional support for this hypothesis comes from the reduction in NOx by regular exercise. Under normal circumstances, the potent vasodilator NO is released during the conversion of L-arginine to L-citrulline. This reaction is catalysed by NOS. However, under certain circumstances, NOS can also produce superoxide (O2*^−^), which reacts avidly with vascular NO^·^ to form peroxynitrite (ONOO^−^). This metabolite is capable of impairing NOS dimerization and function [8, 49], which is called NOS-uncoupling. Such an uncoupling of NOS can occur in the absence of either L-arg or BH4, and increased concentrations of ADMA, a competitive inhibitor of NOS. In the present study, HFD and regular exercise increased h-arg, another substrate of NOS for the production of NO. Although h-arg competes with L-arg for NOS-binding sites, it seems to be a less efficient substrate for NO synthesis. According to März et al., the role of h-arg in NO metabolism is still insufficiently understood [50]. Some studies support the hypothesis that h-arg might increase arginine bioavailability by inhibition of the enzyme arginase, which competes with NOS for the utilization of the key substrate L-arg [50, 51]. If this is correct, the substantial increase in h-arg is likely to exert a protective effect under HFD. This concept is supported by a previous study showing that h-arg supplementation ameliorates blood glucose in mice on HFD [52]. The reduction of ADMA and SDMA observed in the present study further supports the beneficial effect of h-arg in HFD animals. However, h-arg competes with L-arg in more than one way. They both utilize the same transport system for cell entry, and high extracellular h-arg concentrations will result in reduced L-arg uptake [53]. This might at least partially explain the exercise-induced reduction of NOx. The inverse relationship between oxLDL and NOx strongly supports the concept of NOS uncoupling in exercising animals. Considering that NOx levels were lower in non-exercising HFD animals than in non-exercising ND animals, HFD consumption seems to have an independent NO-reducing effect, which is amplified by regular exercise. The very low NOx concentration in exercising HFD animals further supports the concept of an additive NO-reducing effect of HFD and exercise.

In this project, we also investigated the influence of regular exercise on the adipokine, cytokine and chemokine profile in both dietary groups. Obesity is typically associated with an increased secretion of adipokines and a mild tissue inflammation [36]. As expected, our HFD animals showed markedly higher leptin and adiponectin serum concentrations than ND animals regardless of exercise, whereas IGF-1 and resistin were comparable in both dietary groups. Insulin was not considered as blood was collected non-fasting. Exercise was effective in reducing leptin in ND, but not HFD, animals suggesting that the HFD overwhelmed the effect of exercise. The mild increase in serum adiponectin in exeHFD animals might simply reflect the weight loss in these animals. Interestingly, the HFD used in this study had only minor pro-inflammatory effects inducing increased serum concentrations only for MCP-1 and IL-5. Other pro-inflammatory cytokines, such as TNF-α, IFN-γ or IL-6 were comparable between the two dietary groups.

Consistent with previous studies [26, 54], our exercise protocol significantly reduced the serum concentrations of TNF-α, IL-1β, IL-2, MCP-1, MCP-3 and RANTES in HFD animals. MCP-3 and RANTES were also decreased in the exeND group. Both chemokines regulate the migration and infiltration of monocytes and macrophage into solid tissues [33]. The present results are in line with recent findings from Rocha-Rodrigues et al. showing that regular physical activity reduces inflammation in response to HFD administration [26]. Borst et al. have shown that in the context of obesity, visceral fat derived resistin, TNF-*α* and several other interleukins contribute to insulin resistance [35], whereas weight loss or visceral fat removal decrease serum IL-6 and increase the insulin sensitizing hormone adiponectin [34, 35]. The exercise-induced increase of IP-10, IL-12, and MMP activity in ND animals might reflect a mild activation of cellular immunity and tissue remodelling in response to exercise, which is masked in the HFD animals.

The present study has several strengths and limitations that should be considered when interpreting the results. The rather long intervention period with sufficiently sized groups allows robust conclusions about the effects of the different diets and physical activity as important modifiable lifestyle factors that impact CVD risk. Furthermore, our exercise protocol was well controlled and imitated a realistic activity regimen that would normally be considered as healthy. Another strength of this study is the use of validated and strictly quality controlled methods for the measurement of serum lipids. The unlimited access of the animals to food allowed them to compensate the exercise-related increase in energy expenditure through higher food consumption. Due to the unlimited access to food, we cannot account for natural occurring differences in food intake. Furthermore, we lack metabolic studies and blood collections were performed in a non-fasting state. Although this may have an impact on the results of several metabolic biomarkers, we intentionally decided to perform non-fasting blood collections to avoid unwanted psychological stress in the animals. It should also be mentioned that our method for the measurement of TG does not distinguish between TG and free glycerol. However, the blood concentration of free glycerol is < 1 mg/dL, which accounts for approximately 10 mg/dl of triglycerides [55]. Considering that in the SD rats used in this study TG concentrations ranged between 100 and 400 mg/dl, the unintentional detection of free glycerol does not represent a relevant confounder of our results.

Another weakness of our study is the lack of mechanistic information on the effects of exercise on plasma lipids. Considering that body weight was strongly correlated with TG, it can be speculated that the higher fatty acid intake in HFD animals, was the main driver of differences in plasma TG. The HFD used in this study contains approximately 60% of SFA and MUFA, which are incorporated into TG. Exercise instead, induced only minor differences in body weight and plasma lipids. This is not surprising, as our exercise regimen was rather moderate. Previous studies have shown that exercise induces beneficial effects on plasma lipids and lipoprotein lipase activity only above a certain threshold of energy expenditure [56, 57]. Another putative mediator of plasma lipids could be adiponectin, which is known to increase energy expenditure through fatty acid oxidation in target organs, such as liver and skeletal muscle [58]. This theory is supported by the inverse correlations of TC and HDL-C with adiponectin.

## Conclusion

In summary, long-term moderate exercise may alter the delicate equilibrium between oxidative and anti-oxidative compounds leading to an uncoupling of NOS with higher oxLDL and lower NOx concentrations.

However, these metabolic effects do not necessarily compromise the beneficial reduction of pro-inflammatory cytokines, such as TNF-*α*, IL-1*β*, IL-2, MCP-1, MCP-3 and RANTES. This conundrum adds to the controversial role of oxLDL in pathologic developments like atherosclerosis and CVD and should trigger additional research that helps to understand lipoprotein oxidation and NO production in response to exercise and different dietary habits.

## Data Availability

All data and materials as well as software application or custom code support their published claims and comply with field standards.
